# Approaching Avoidance: A Step Essential to the Understanding of Craving

**Published:** 1999

**Authors:** Mary Jo Breiner, Werner G. K. Stritzke, Alan R. Lang

**Affiliations:** Mary Jo Breiner, M.S., is a doctoral student working under the supervision of Dr. Lang in the Department of Psychology at Florida State University, Tallahassee, Florida. Werner G. K. Stritzke, Ph.D., completed his doctoral work with Dr. Lang and is now an assistant professor of psychology at the University of Western Australia, Perth, Australia. Alan R. Lang, Ph.D., is a professor of psychology at Florida State University, Tallahassee, Florida

**Keywords:** AOD (alcohol and other drug) craving, alcohol cue, avoidance conditioning, theory of AODU (alcohol and other drug use), causes of AODU, predictive factors, classical conditioning, AOD sensitivity, expectancy, motivation, causal model, AOD prevention, intervention, behavior modification, risk assessment, literature review

## Abstract

Craving is only one component of the mental processes that influence drinking behavior. Alcohol-related cues (ARCs) can set in motion a dynamic competition between inclinations to approach drinking and inclinations to avoid drinking. Craving can thus be integrated into a comprehensive model of decisionmaking in which ambivalence or conflict is a key element. The relative strength of each component of the ARC reaction can fluctuate over time as well as in response to both subjective states and environmental circumstances. Simultaneously and independently evaluating these opposing responses puts clinicians in a better position to influence the relative weight that the patient assigns to the positive and negative outcomes of alcohol consumption.

Most researchers and practitioners in the alcohol field agree that alcohol “craving,” defined here as an inclination to approach and consume alcoholic beverages, is a critical feature of alcohol use disorders. Such craving may be activated by stimuli or “cues” (see sidebar by Tiffany, p. 216) that the patient has come to associate with reinforcement from drinking. However, an exclusive focus on the forces attracting a person toward alcohol consumption is arguably too restrictive and contrasts sharply with the thrust of many traditional treatment strategies, which tend to minimize consideration of the rewarding aspects of excessive drinking. Practitioners typically strive to foster abstinence or reduced drinking among problem drinkers by advocating avoidance and restraint. Accordingly, the transformation of alcohol-relevant cues into signals warning of impending punishment is a tactic often used by practitioners to emphasize the adverse consequences of drinking.

The goal of this article is to encourage both scientists and clinicians to appreciate the complexity of responses elicited by alcohol cues, particularly the likelihood that these cues can prompt a dynamic competition between inclinations to *approach* drinking and inclinations to *avoid* drinking. By simultaneously and independently evaluating these opposing responses, researchers and treatment professionals might understand the essentially unidimensional construct of craving better and integrate it into a comprehensive motivational model.

The concurrent operation of both approach and avoidance inclinations in people experiencing problems with alcohol and other addictive substances is not a new idea. Indeed, the significance of these competing motives is apparent in current diagnostic criteria for addiction (i.e., dependence) to alcohol or other drugs (AODs). These criteria include using the substance in larger amounts or over a longer period than was originally intended, along with a desire for the substance despite efforts to cut down or control its use (American Psychiatric Association 1994). Based on these characteristics, AOD-dependent patients are seen as both drawn toward and repelled from substance use.

This observation has led a number of theorists (e.g., Orford 1985) to identify ambivalence or conflict as a key element of excessive appetites of many kinds. Thus, an adequate theory of alcohol use problems must explain not only why alcoholics return to drinking despite resolutions not to do so but also why they often succeed, either temporarily or permanently, in refraining from problem drinking. Heather (1998) has argued that addictive behavior is defined, at least in part, by ambivalence associated with the decisionmaking process. Intervention strategies consistent with this concept attempt to motivate recovery by influencing the relative weight the patient assigns to the positive and negative outcomes of alcohol consumption (Prochaska et al. 1997; Miller and Rollnick 1991).

The theorized role of ambivalence in alcohol use disorders suggests that craving is only one component of a multidimensional phenomenon comprised of largely independent inclinations to approach and avoid drinking. This framework for understanding responses to alcohol-related cues (ARCs) assumes that the relative strength of each component of the reaction can fluctuate over time as well as in response to both subjective states and environmental circumstances. Such a conceptualization departs from the traditional view that craving alone drives decisions about drinking. However, it does incorporate mechanisms by which low-intensity, seemingly “irrelevant” stimuli, thoughts, and actions can set the stage for later inclinations to approach and consume alcohol (Marlatt and Gordon 1985). This article strives to integrate the concept of craving into a comprehensive model that better captures the reality of addicts’ struggles along the dual pathways of indulgence and restraint. A recurrent theme is that responses to alcohol-relevant cues are multifaceted and dynamic.

**Figure:**
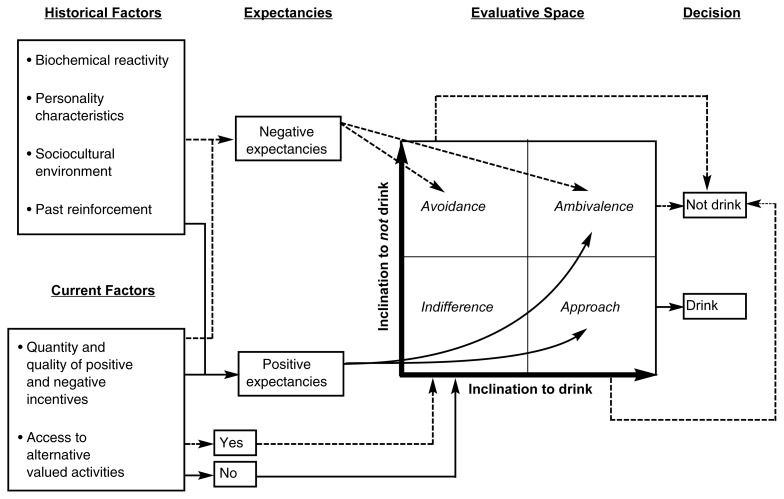
A multidimensional model of inclinations to drink or not drink. Dashed lines represent factors that promote alcohol avoidance, whereas solid lines represent factors that promote the desire to approach alcohol. This table depicts only the most essential connections with regard to historical factors, expectancies, motivations, and decisions in alcohol use, although other connections may exist.

## Pathways to Indulgence and Restraint

Many modern motivational theories of alcohol use rest on the premise that problem drinking is mediated by the same decision processes that govern all alcohol use and that people essentially choose between drinking and alternative actions. According to this view, people decide whether to consume alcoholic beverages by comparing the positive consequences they expect to experience by drinking with those they expect from not drinking. The figure on page 198 illustrates the parallel nature of the pathways that promote either indulgence or restraint. This figure adds two features to a model proposed originally by Cox and Klinger (1988): (1) it incorporates a complementary perspective, based on behavioral theories of choice (Vuchinich and Tucker 1998), which holds that preference for alcohol is inversely related to the accessibility of alternative valued activities, and (2) more importantly, it incorporates an “evaluative space” to represent the intersection of the opposing pathways of approach and avoidance. Such ambivalent or conflicting inclinations appear to be central both to cognitive-processing theories of craving (Tiffany 1990) and to recent shifts in thinking about the essence of addiction in more general terms (Heather 1998) (see the articles in this issue by Anton, pp. 165–173, and by Tiffany, pp. 215–224). The integration of a variety of competing factors and responses—both positive and negative—within a multidimensional evaluative space ultimately determines a person’s choice to drink or not drink, thereby underscoring the potential importance of the evaluative space and emphasizing the need for an explicit assessment of the two independent dimensions.

### The Role of Historical Factors

Influences termed “historical factors” contribute to the decisionmaking process (Cox and Klinger 1988). Such factors can shape a person’s drinking experiences and his or her subsequent inclination to consume alcohol. For example, genetically based aspects of individual biochemical reactivity can contribute to intrinsically pleasant (e.g., euphoric) or unpleasant (e.g., flushing) effects precipitated by alcohol consumption (Newlin and Thomson 1990). The strength of certain personality characteristics, such as antisociality (e.g., a tendency toward aggressive or criminal behavior) and sensation seeking (e.g., a strong predilection for novel and risky experiences), have also been implicated in the associated risk for alcoholism (Lang 1983). External factors, such as sociocultural drinking norms and personal experiences with alcohol-related consequences, can also support, inhibit, or modulate the use of alcohol and influence drinking behavior (White 1993).

Thus, biochemical reactivity, personality characteristics, the sociocultural environment, and personal experience of drinking outcomes help determine a person’s response to ARCs. However, the above-mentioned factors are not necessarily internally consistent or static. Consequently, a person’s responses to ARCs often represent a fusion of attractions and repulsions, both within a particular set of circumstances and across time. This can, of course, contribute to ambivalence.

Excessive drinkers tend to develop more marked “conditioned” responses to cues (e.g., the smell of alcohol or perhaps a certain mood) (see sidebar by Tiffany, p. 216) that have been repeatedly associated with drinking. In other words, exposure to such cues can elicit physiological, emotional, and cognitive reactions, including those that constitute “craving.” Several influential conditioning models have been advanced to explain the relationships among ARC exposure, craving, and subsequent alcohol consumption, particularly in alcoholics. These models have been extensively reviewed elsewhere in the literature (Drummond et al. 1995); a full description of each of these models is beyond the scope of this article.

However, all conditioning models posit that ARCs ultimately elicit a craving response that motivates further alcohol use. Each such model accounts for approach inclination (i.e., craving) in terms of the action of two interrelated learning processes: (1) the association of previously neutral stimuli (e.g., a pub sign) with alcohol consumption and (2) the subsequent connection of these cues with certain reinforcing actions of alcohol that encourage future indulgence in drinking. Such models diverge from one another mainly with respect to the mechanisms by which alcohol cues are presumed to motivate alcohol use. In particular, each model offers different descriptions of the reactions elicited by alcohol cues.

Some conditioning models (Wikler 1948) hypothesize that ARCs stimulate an aversive state (e.g., elicit subclinical withdrawal symptoms or negative emotional responses associated with deprivation) that lies behind craving and the desire to drink. Other conditioning models (Stewart et al. 1984) suggest that alcohol cues stimulate an appetitive state by signaling access to desirable effects (e.g., euphoria) through drinking. In the first instance, craving or motivation to drink is described as a desire to obtain relief from an aversive state (i.e., negative reinforcement); in the second instance, craving is viewed as a desire to experience the pleasurable effects of alcohol intoxication (i.e., positive reinforcement). A hybrid model (Baker et al. 1987) involves speculation that cues can elicit both types of motives or craving. In any event, these models predict a similar outcome: Exposure to ARCs should increase the inclination to “approach” alcoholic beverages.

Although these models have provided a rich theoretical foundation for the initial study of craving and other reactions to alcohol cues, they do not address the potential elicitation of avoidance inclinations by the same cues. Moreover, no existing model can adequately account for all of the evidence on craving accumulated thus far. Recent developments in neuropsychopharmacology appear to address at least some of these shortcomings by providing potentially important building blocks for the construction of a more complete picture of the development and operation of craving. In particular, advances in this arena have yielded valuable information about the specific effects of AODs and AOD-relevant stimuli on brain systems and have offered suggestions about the possible impact of these effects on subsequent behavior.

For example, Robinson and Berridge (1993) have highlighted the importance of “neuroadaptation,” or sensitization, of certain brain systems due to repeated substance use. Their theory takes a step toward the integration of biological and learning processes into a more comprehensive model of craving. This analysis holds that psychoactive substance use, especially by relatively inexperienced consumers, can produce a pleasant affective response by stimulating neural systems associated with reward. This is regarded as a simple “liking” for the effects of the substance and can motivate further use.

Robinson and Berridge (1993) further posit that the reinforcing effects of AODs do not *maintain* long-term substance use by addicts. Rather, these two researchers assert and provide evidence suggesting that the more compulsive “wanting” of the substance (i.e., craving) may result from repeated substance use that provokes a specific neuroadaptation—that is, the progressive and persistent hypersensitization of the dopamine pathways (see the article in this issue by Anton, pp. 165–173) implicated in the mediation of wanting or craving the substance. The theory specifically segregates the mechanisms responsible for “liking” (based on simple positive reinforcement) from the mechanisms underlying “wanting” (sensitization to cues associated with the substance).

This distinction enables the model to account for the continued use of AODs even when the subjective pleasure derived from them has disappeared or diminished greatly, as is often the case for addicts. A key element of this model is its proposition that the hypothesized neuroadaptation, or sensitization, is influenced by associative learning processes in such a way that exposure to cues which have been reliably paired with substance use enables the cues themselves to stimulate sensitization and thereby increase and sustain “wanting” for the substance. Hence, craving is viewed as involving both biochemical and learning processes.

Despite these insights, the theory of neuroadaptation in craving, like the earlier conditioning models of reactivity to alcohol cues, can be faulted for its exclusive focus on the development and elicitation of approach inclinations. Given the high likelihood that excessive drinkers will have histories that include punishment as well as reinforcement from their extensive alcohol use, they should harbor both negative and positive associations with alcohol cues. Not only should this dual association lead to a certain amount of ambivalence in their response to alcohol cues, but it should also raise questions about what neural substrates might underlie the development and elicitation of avoidance inclinations or motives to not drink.

One solution to this dilemma is to consider the possibility that in addition to mediation by appetitive brain systems such as those referred to in connection with neurobiological models of craving, response to alcohol cues and decisions to not drink may involve a parallel, aversive brain system that governs response to threats and other negative stimuli. Indeed, substantial evidence indicates that such a system exists (Gray 1987; P. Lang 1995), and certainly its activation by alcohol-related cues is plausible.

Moreover, much recent research indicates that although both the appetitive and aversive systems are subcortically based,^1^ they can interact with more complex cognitive processes,^2^ including attention, perception, imagery, and certain types of memory (LeDoux 1995). Not only do these relatively independent systems provide a neural basis for ambivalence, but their interactions with the cortex also point to the complementary roles of both simple conditioning and higher level cognitive processes in reactions to alcohol cues and in inclinations to drink or not drink. However, before elaborating on how competing associations and cognitions might influence the approach or avoidance of alcohol, this article briefly examines what Cox and Klinger (1988) called “current factors” and the role they might play in shaping subsequent decisions to drink or not drink.

### The Role of Current Factors

Current factors are variables in the immediate situation that influence whether a person is inclined to approach or avoid drinking. For example, to the extent that alcohol is available, the context can be seen as conducive to drinking. Similarly, if other people around the target person encourage drinking, he or she is more likely to follow the path of approach than the path of avoidance. Conversely, to the extent that alcohol is unavailable, the immediate context is not appropriate for drinking, and/or those around the target person discourage drinking, the person is more likely to avoid, than approach, alcohol. These immediate circumstances are considered when people assess whether the consequences of drinking or not drinking will likely be positive or negative. In addition to situational factors such as these, the availability of alternative behavioral options with predictable outcomes can mediate the impact of the current situation on decisions to drink or not drink.

According to behavioral theories of choice (Vuchinich and Tucker 1998), preference for alcohol consumption varies depending on access to other valued and enjoyable activities. The availability of alternative rewarding activities promotes an indifferent “take it or leave it” attitude toward alcohol and predicts that people with such options will more likely follow the path of avoidance than that of approach. However, if alternative sources of reward are unavailable or limited, or if access to them is delayed or requires more effort, then a person is more likely to choose to drink rather than not drink (Vuchinich and Tucker 1998).

In this context, many of the potential benefits associated with not drinking (e.g., avoiding a hangover) seem particularly distant when choices are made, thereby diminishing their impact on the decisionmaking process. This fact enhances the relative impact of the more immediate rewards associated with indulgence.

### The Role of Expectancies

Alcohol outcome expectancies (i.e., beliefs that people hold regarding the behavioral, cognitive, and emotional effects of alcohol consumption) represent a third category of variables that influence the likelihood that a person will be inclined to either approach or avoid alcoholic beverages. Such expectancies are shaped by the positive and negative consequences that a person has experienced as a result of drinking. However, the fact that beliefs about alcohol effects develop in children and adolescents well before they engage in any drinking attests to the strong impact of social and observational learning on them (Dunn and Goldman 1998; Lang and Stritzke 1993).

Regardless of the origin of alcohol expectancies, and the recognition that expectancies can change across time and context, certain obvious predictions can be made based on aggregated expectations. To the extent that alcohol effects are believed to be mainly positive, and potential negative outcomes are viewed as relatively modest, people should be more likely to follow the path of approach than avoidance. Furthermore, this finding should be particularly true when anticipated reinforcements are immediate and punishments delayed. Conversely, to the extent that the effects of drinking are expected to be predominantly negative, even when some expectation of modest positive effects exists, people should ordinarily be motivated to avoid, rather than approach, alcohol. However, such applications of global expectancies may overlook the relevance of alcohol beliefs specific to particular situations and often fail to fully consider the independent roles of divergent expectancies.

A critical question emerging from alcohol expectancy research as it pertains to predictions about people’s choice to drink or not drink concerns the relative contribution of positive versus negative expectancies (Jones and McMahon 1998). As was the case with craving research, most of the early studies of alcohol expectancies focused on beliefs that would attract one to drinking (i.e., foster approach inclinations). In other words, the research highlighted the importance of positive expectancies to understanding alcohol use and its problems. Pertinent evidence suggests that the more positive expectancies people hold regarding alcohol, the more likely they are to use alcohol and, if they already drink, the more likely they are to report higher levels of alcohol consumption (Lang and Michalec 1990).

However, in investigations focusing on negative expectancies, the findings suggest a more dynamic relationship between negative expectancies and alcohol consumption (Jones and McMahon 1998). For example, negative expectancies in both light and moderate drinkers seem to be associated with less alcohol use, perhaps indicating that negative expectancies promote avoidance and restraint. Because negative expectancies among lighter drinkers may be relatively mild (e.g., “I would expect my handwriting to be affected” or “I would expect to feel fuzzy”), positive expectancies may remain dominant. However, people are more likely to experience weightier negative consequences as their drinking escalates or persists (e.g., “I would expect to get into a fight” or “I would expect to lose my job”). This finding suggests that negative expectancies might ultimately challenge initially dominant positive expectancies and subsequently promote ambivalence toward drinking. However, until the point is reached where negative expectancies begin to affect drinking behavior, a person may experience a period during which negative expectancies and alcohol use increase simultaneously (Jones and McMahon 1998).

In their review of studies investigating the concurrent operation of both positive *and* negative alcohol expectancies, Jones and McMahon (1998) make a compelling case for precisely this point. They present evidence for the pivotal role that negative beliefs about alcohol effects and outcomes play in determining decisions about drinking. Their conclusion is consistent with the proposal that much may be gained from considering the avoidance inclinations that compete with craving and other approach inclinations to influence the choice to drink or not drink.

Holding particular beliefs about alcohol’s effects is not in and of itself a condition sufficient to cause drinking to occur (Leigh 1990). People must also value the consequences they expect. In this regard, strong empirical evidence indicates that subjective evaluations of the desirability of expected outcomes linked to alcohol use moderate the relationship between expectancies and drinking decisions (Jones and McMahon 1998). According to motivational models of alcohol use, subjective evaluations of expected consequences of indulgence provide motives for drinking that are the more proximal determinants of choices about alcohol use (Cox and Klinger 1988).

### The Role of Motives

The evaluative space of the model depicted in the figure on page 198 is useful when considering a patient’s motives or reasons for drinking or not drinking alcohol. Research in the area of motivation to drink has not ordinarily been designed to analyze competing inclinations to either approach or avoid alcoholic beverages, nor has it considered changes in motives across time and context. However, research has revealed that different global motives for drinking are associated with fairly distinct patterns of alcohol use and abuse (Cooper 1994). For instance, efforts to enhance pleasure and to cope with negative emotions have both been identified as potentially important motives for alcohol use. Drinking to cope with negative emotions, however, is primarily predictive of alcohol problems in adolescents (Cooper et al. 1995) and alcohol dependence in adults (Carpenter and Hasin 1998). Unfortunately, distinct biases analogous to those observed in alcohol-expectancy research are also evident in the extant literature on alcohol-related motives. Clearly, the focus has been primarily on the reasons why people say they want to drink, rather than on the potentially important reasons why people might want to avoid alcohol and choose to not drink.

As a first step toward redefining the focus of alcohol research, we developed a questionnaire to assess motives for *not* drinking alcohol. This questionnaire was based on the general principles of an expanded version of Cox and Klinger’s (1988) motivational model of alcohol use and applied a measurement approach similar to that used by Cooper and colleagues (1995) to study motives for drinking.

Preliminary results indicate that at least among adolescents, different motives for not drinking are strongly linked to different aspects of alcohol use. For example, the frequency of alcohol use and the category of drinker (i.e., drinker versus abstainer) are predicted by constraints associated with religion and family and by motivational indifference. In contrast, the quantity of alcohol consumed on a typical drinking occasion is predicted only by fear of negative consequences.

These findings emphasize the importance of studying people’s motives for avoiding alcohol and suggest that both alcohol education and prevention programs for teenagers should be tailored accordingly. If a program’s objective is to prevent or forestall young people’s initiation to drinking, an effective strategy might be to emphasize traditional relationships and encourage involvement in rewarding activities that are alternatives to drinking and thereby increase indifference toward alcohol. However, if the objective is to reduce alcohol consumption among drinkers, the most effective approach might be to focus on the adverse consequences of indulgence, perhaps even threatening or enforcing stronger negative sanctions.

Each of the steps and diverse categories of variables reviewed thus far involves competing forces. These forces, in turn, must be weighed and combined to determine whether the decisional balance will tip toward drinking or not drinking for any one person in any situation. In other words, a full understanding of the impact of craving on alcohol use requires consideration of the relative impact of the inclination to avoid alcohol use as well.

## The Concept of Ambivalence and the Mapping of an Evaluative Space

In his treatise on why “excessive appetites” for alcohol consumption and other addictive behaviors revolve around conflict or ambivalence as the central, defining construct, Orford (1985) cited the work of two independent researchers who had applied classic conflict theory (Miller 1944) to the phenomenon of excessive alcohol use. Both Astin (1962) and Heilizer (1964) suggested that for problem drinkers, alcohol-associated cues induce an approach-avoidance conflict. According to both Astin’s and Heilizer’s models, conflict arises in alcoholics because previous alcohol use has been both reinforced and punished. Both authors noted that rather than sustaining an ambivalent state, alcoholics exposed to alcohol cues tend repeatedly to resolve the conflict in the approach direction (i.e., they choose to drink). In other words, their desire to drink appears to increase as they near the goal (i.e., alcohol), whereas their avoidance inclination appears to remain constant or even decline along the way.

Astin (1962) proposed that the mechanism underlying this observed pattern of resolution in favor of approach was related to the timing and nature of the consequences associated with drinking. Positively reinforcing consequences of alcohol use (e.g., the euphoria of intoxication or relief from stress) tend to occur soon after consumption, whereas adverse consequences (e.g., hangover or punishment for failure to fulfill responsibilities) tend to occur later in time. Similarly, Heilizer (1964) proposed that the strengthening of approach inclinations could be attributed to the increasing salience of prospective alcohol reinforcers, relative to punishers, as one gets closer to the goal. Approach stimuli become more prevalent and gain intensity, whereas stimuli related to alcohol avoidance (i.e., punishment cues) tend to remain constant. Heilizer argued that these responses occur because approach cues are likely to involve explicit characteristics of the alcohol-drinking context and its immediate consequences. In contrast, avoidance cues are liable to be more remote and/or abstract (e.g., cognitive representations of future negative consequences) in their associations. Although neither of these theorists—Astin nor Heilizer—actually tested their hypotheses, their work has obvious relevance to the study of reactivity to alcohol cues. Moreover, data accumulated in connection with modern behavioral theories of choice as applied to drinking (Vuchinich and Tucker 1998) tend to support at least Astin’s assertion regarding the importance of temporal factors.

Heather’s (1998) proposal of a three-level conceptual framework for addiction also involves both approach and avoidance components. He regards ambivalence, expressed behaviorally as repeated failures to refrain from substance use despite intentions to do so, as the basis of the definition of addiction. Briefly stated, the three components of Heather’s framework are as follows: (1) persistent desire to use the substance because of its rewarding consequences, (2) neuroadaptation to a substance resulting from repeated use, and (3) a pattern involving the apparent inability of addicts to curtail their indulgence despite trying to do so. The ambivalence central to Heather’s framework represents conflict attributable to interplay between the neuroadaptations from repeated use and the person’s history of consequences from use.

Beyond stressing the importance of the role of ambivalence and possible neural substrates involved in it, what is of particular relevance to the multidimensional framework of reactivity to alcohol cues and decisions about drinking is Heather’s speculation about the cognitive-behavioral mechanisms underlying the development of conflict. Drawing from Ainslie’s (1975) theory of impulsive behavior, Heather observed that when faced with a choice between “early small” and “late large” rewards, addicts repeatedly fail to implement normal cognitive compensation for their “irrational preferences.” In other words, addicts appear to have difficulty basing their decisionmaking on anticipation of *future* outcomes, an ability that ordinarily enables humans to adjust their current behavior in order to obtain larger rewards (or avoid punishment) later.

Although the reasons for this failure are not specified, a basis for speculation does exist. For example, impaired decisionmaking may occur because the level of immediate reinforcement available provokes the brain to adapt to repeated exposures to the psychoactive substances, and the operation of neural systems underlying the inclinations of addicts ultimately begins to deviate from that evident in nonaddicts. In this connection, Heather notes that his perspective is compatible with the neuroadaptation theory of Robinson and Berridge (1993) discussed earlier. Moreover, an explanation for the development of a “desire to curtail indulgence,” so central to Heather’s conflict perspective, can be derived from consideration of associations linking drug cues to addicts’ discomfort with their compulsion and other punishing consequences of repeated use.

Regarding these insights, the use of restraint when faced with the proximal temptations associated with indulgence requires the processing of information about consequences that are often distal and perhaps more abstract as well. Thus, the decision to not drink may be more cognitively demanding than the decision to drink, therefore rendering restraint the relatively more difficult path to follow.

In sum, craving may be best conceptualized within a broader, multidimensional perspective that incorporates the relative influence of an inclination to not drink. This involves a framework in which competing motives are evaluated. The evaluative space indicated in the figure on page 198 depicts this framework, which is described by four quadrants. Craving, in its classic form as intense and unrestrained “wanting” is synonymous with the *approach* quadrant, whereas strong inclination to not drink, in the absence of any significant inclination to indulge, is represented by the *avoidance* quadrant. If both response inclinations are balanced but at a low level of intensity, a person is characterized by *indifference* about drinking alcohol, whereas if both inclinations are balanced and at a high level of intensity, a person struggles with *ambivalence* about choices to drink or not drink.

## Evidence for a Multidimensional, Ambivalence Model

A small but growing number of empirical findings support the view that an investigation of a multidimensional or ambivalence model of choices about drinking may be an important step toward better understanding the relationship between craving and substance use. Greeley and colleagues explicitly acknowledged the potential of alcohol-related cues to elicit avoidance inclination (Greeley et al. 1993*a*; Greeley et al. 1993*b*). They used a bidirectional “craving scale” to measure alcoholics’ and social drinkers’ subjective reactions to alcohol cues relative to neutral cues. At one extreme of this scale was “definitely do not want a drink of alcohol,” whereas at the other end of the scale was “an extreme desire for a drink of alcohol.” Unfortunately, because the researchers attempted to assess approach and avoidance by means of a single scale, participants were required to collapse the two inclinations and arrive at a “sum.” The authors acknowledged that this summation process obscured measurement of the true level of ambivalence that participants may have experienced. Consequently, the authors called for future studies to measure approach and avoidance inclinations independently.

Avants and colleagues (1995) sought to examine the reactivity of addicts to drug cues using separate assessments of approach (i.e., “craving”) and avoidance (i.e., “aversion”) inclinations. The participants, who were on methadone maintenance for heroin addiction while undergoing treatment for cocaine dependence, viewed a videotape depicting persons using cocaine. The participants also were asked to handle their preferred type of cocaine paraphernalia. Before and after exposure to these cues, the participants were asked to use rating scales to respond to the following questions: “How much do you crave cocaine right now?” and “How much does the idea of using cocaine turn you off right now?” Results indicated that overall craving and aversion ratings were negatively correlated at baseline, but they were not significantly correlated after cocaine cue exposure, suggesting that these inclinations vary independently of one another.

Further analysis revealed the presence of four subsamples of patients demonstrating differing response patterns to the cues. One group showed an increase in craving and a decrease in aversion, indicating a clear shift toward the *approach* quadrant of the evaluative space. Another group showed an increase in craving but no decrease in aversion. Depending on the initial level of aversion, their craving may or may not have been balanced by an equivalent level of aversion, and thus they could be represented in either the *approach* or the *ambivalence* quadrant. A third group showed no increase in craving, but a decrease in aversion, indicating a shift away from the *avoidance* and toward either *approach* or *indifference,* depending on the initial level of craving. Finally, a group of “nonresponders” showed no increase in craving and no decrease in avoidance.

When analyzed together, these results indicated that levels of craving and aversion, constructs that can be readily mapped onto the dimensions of the evaluative space, could be altered by exposure to substance-related cues. Moreover, evidence suggests that these changes not only varied independently, but could also be influenced by individual differences. For example, further analyses revealed that individual differences in two areas distinguished nonresponders from those who showed the most pronounced shift toward the *approach* quadrant. Relative to nonresponders, those who reported an increase in craving and a decrease in aversion also perceived cocaine to be more reinforcing and less punishing, and they saw themselves as less able to avoid using cocaine in certain high-risk situations. Thus, the work of Avants and colleagues (1995) provided general support for a multidimensional, ambivalence model of reactivity to drug cues and helped establish the model’s clinical relevance by noting that observed shifts appeared to be associated with variables such as perceptions of the net benefit of drug taking and of self-efficacy in coping with high-risk situations.

More direct evidence in support of this model stems from research conducted in our own laboratory (Breiner et al. 1997). Using a large sample of undergraduate students, we measured separate approach and avoidance reactions to photographic stimuli depicting several kinds of consumable substances, including alcohol and cigarettes. Respondents with various patterns of routine usage of these substances were asked to view slides, responding after each one to the following questions: “How much do you want to *consume* the item pictured in the slide?” and “How much do you want to *avoid consuming* the item pictured in the slide?” Whereas ratings for approach and avoidance were significantly negatively correlated in abstainers from both categories (nonsmokers and nondrinkers), results for light and moderate alcohol drinkers and for occasional and daily smokers indicated no significant correlations between approach and avoidance. This independence of variation argues for the need to separate these two dimensions and for the potential of the competing inclinations to coexist.

Data from a college student sample also yielded some particularly interesting information on reactions to cigarette-relevant stimuli as a function of desire to change behavior. Participants were divided into three groups: nonsmokers, regular smokers not trying to quit, and regular smokers currently trying to quit. We then compared the participants’ approach and avoidance reactions to cigarette stimuli. Results indicated that nonsmokers were characterized by a combination of low approach and high avoidance inclinations, representing the *avoidance* quadrant in the model. In contrast, regular smokers not trying to quit reported high approach and low avoidance inclinations, placing them in the *approach* quadrant in the model. Most interesting was the finding that smokers who were trying to quit reported high approach and high avoidance inclinations characteristic of the *ambivalence* quadrant. Thus, it was not the level of craving, but rather avoidance, that identified smokers who were ready to change.

In addition to evidence from research on reactions to drug cues, data from studies investigating memory processes are consistent with a multidimensional or ambivalence perspective. For instance, Leigh and Stacy (1998) reported that memory associations related to both reinforcing and punishing consequences of alcohol use can be activated by the same type of cognitive task. They examined associative memory and alcohol use and demonstrated that participants’ histories of alcohol use (i.e., quantity and frequency of drinking) predicted their associative memory responses to both positive and negative outcomes of drinking. When given a list of positive and negative outcomes not specific to alcohol (e.g., “feeling good”; “forgetting problems”; and “being more social” versus “feeling sick,” “being depressed,” and “losing control”), heavy social drinkers generated significantly more alcohol-specific responses for both types of outcomes than did light social drinkers. This finding further supports the notion that approach or craving should be integrated with avoidance to reflect the multidimensional nature of responses that seem especially likely to accrue as repeated alcohol use strengthens associations in memory that link alcohol cognitions to both positive and negative consequences of drinking.

Strong memory associations to alcohol-related cues and behaviors are also central to Tiffany’s (1990) influential cognitive processing model of craving and substance use. According to this model, if practiced regularly, drug use becomes automatized and, like other highly practiced skills, relies on strong memory associations for rapid and effortless execution.

Within the framework of the evaluative space, this response would be characteristic of the craving associated with the *approach* quadrant. However, Tiffany maintains that craving does not involve automatic processing. In his model, craving refers to a constellation of responses supported by nonautomatic, effortful cognitive processes activated only if the habitual sequence of drug use behaviors is blocked by limited access to the substance or by an intentional effort to curtail use. Implicit in at least the latter of these scenarios is the experience of ambivalence, operationalized in the model as the simultaneous activation of opposing response inclinations. To what extent can the two views be reconciled?

One perspective on cognitive conflict or ambivalence provides for the explicit definition and measurement of component dimensions, whereas conflict in Tiffany’s cognitive processing model is inferred from the increased cognitive effort thought to be associated with the dual processing required when competing response inclinations are present (Cepeda-Benito and Tiffany 1996; Sayette et al. 1994). However, the two models are complementary in that Tiffany’s distinction between the automatic processing associated with direct, unimpeded approach and the effortful processing associated with craving is also a distinction that differentiates the quadrants in the multidimensional, ambivalence model.

Preliminary laboratory evidence is pertinent here (Stritzke et al. 1997). Participants who simultaneously reported high levels of both approach and avoidance inclinations in connection with the viewing of appetizing food slides showed a significant increase in heart rate during slide viewing. Such an elevation of heart rate is consistent with the engagement of greater cognitive effort during the viewing of food slides (Hare 1972). In contrast, participants who rated their reactions to the same slides as high in approach but low in avoidance showed a decrease in heart rate. This decrease in heart rate reflects simple orienting but no elaborative processing (Graham and Clifton 1966). Using similar picture-viewing protocols, comparable contrasts in heart rate responses were also found for restrained (i.e., ambivalent) eaters versus nonrestrained eaters (Stritzke et al. 1997) in a combined sample. These data support the applicability of a multidimensional, ambivalence model to a wide range of addictive and habitual behavior problems.

## Summary and Implications for Prevention and Intervention

This article began by noting that the traditional focus of alcohol research on craving as the force driving individuals down the path toward alcohol consumption fails to adequately account for the role of competing inclinations to avoid alcohol and not drink. A similar bias also exists in research on “historical” and dispositional risk factors as well as on situational or “current” factors relevant to choices about drinking. A brief review of recent research developments, especially in the areas of alcohol expectancies and drinking motives, has further revealed that avoidance inclinations associated with the pathway of restraint are potentially important determinants of the choices to drink or not drink. Within this context, evidence indicates that factors promoting avoidance inclinations appear to be better predictors of treatment outcome than factors promoting approach inclinations. Clearly, the full picture involves more than the stimulation of craving by cues that have been associated with drinking.

Reactions associated with alcohol cues are multifaceted and capable of interacting with a wide range of other factors to touch off parallel and potentially contradictory response chains that must be resolved. Recognition of these complexities and a shift toward simultaneous consideration of competing approach and avoidance inclinations are essential to a better understanding of craving and choices about drinking. A multidimensional, ambivalence model provides a framework for future investigations in this area as well as suggests avenues for prevention of drinking by young people and for the treatment of anyone who suffers from problem drinking.

Most prevention programs emphasize the adverse consequences of drinking and promote abstinence from all drinking, leaving children to wonder why anyone would drink or how they could drink moderately and responsibly under appropriate circumstances as adults (Lang and Stritzke 1993). However, societal ambivalence about alcohol is liable to be reflected in the ambivalence many young people experience as they face the challenge of making responsible decisions about drinking. Evidence suggests that developmental shifts occur in the way children evaluate alcohol’s positive and negative effects (Dunn and Goldman 1998). For prevention strategies to be optimally effective, researchers need to understand how problem drinking does *not* develop and what produces protection against it (Zucker and Gomberg 1986). Utilizing a framework that accounts for the balance between approach and avoidance inclinations is an important step in that direction.

When considering interventions for people with drinking problems, every clinician knows that motivation is a vital element. Consequently, an initial and fundamental goal of contemporary motivational interviewing techniques is to provoke clients to recognize a “discrepancy” between their important personal goals and the harm stemming from their strong inclination to drink (Miller 1998). By encouraging an assessment and weighing of the pros and cons of drinking versus not drinking, the aim is to strengthen a client’s inclination to avoid alcohol relative to the inclination to approach it. In terms of the concept of an evaluative space, “discrepancy” has been achieved when a client moves from the *approach* quadrant into the *ambivalence* quadrant. When working with clients who experience addictive behavior problems, the application of an ambivalence model, with its two-dimensional evaluative space, has significant advantages over unidimensional assessments of craving. Clients who know that the strength of their inclination to not drink will also be measured seem more able to acknowledge their inclination to drink. This works to improve the validity of self-reports because it diminishes the demand to deny craving that is often so intense in clinical settings.

Furthermore, ambivalence can be a normal and important step toward increasing readiness and maintaining efforts to change. In fact, to the extent that clients struggle with strong inclinations to drink alcohol, ambivalence may be the only buffer between the resolve not to drink and relapse during the initial stages of treatment. In this connection, we wish to emphasize that ambivalence is associated with *inaction,* whereas drinking is a state of *action.* As long as ambivalence is maintained, lapses into drinking should be minimized. It also follows that fluctuations in the strength of avoidance inclinations may be better predictors of treatment outcome than the strength of approach inclinations, which often remain high and fairly constant, at least through the early phase of intervention. Tiffany (1990) has pointed out that craving alone is not necessary for substance use. However, according to a more integrative and comprehensive analysis, when craving occurs, it must be counterbalanced by avoidance inclinations that often demand intense cognitive effort to produce ambivalence if substance use is ultimately to be restrained.

## Footnotes

1 The inner (i.e., subcortical) regions of the brain are primarily involved in sense perception, motor coordination, emotion, and the unconscious aspects of reinforcement. The brain’s outer layer (i.e., cortex) organizes and interprets the totality of sensory information and plans motor activity.

2 Cognition is the process of obtaining, organizing, and using intellectual knowledge.
