# Erratum

**Published:** 1999

We regret that an error occurred in the reproduction of the figure included in the article “Approaching Avoidance: A Step Essential to the Understanding of Craving,” by Mary Jo Breiner, Werner G. K. Stritzke, and Alan R. Lang, in *Alcohol Research & Health* volume 23, number 3, page 197. The corrected figure and the full figure legend appear below.

**Figure:**
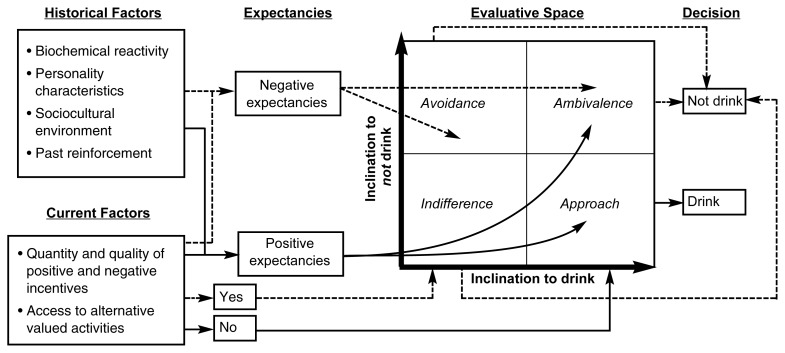
A multidimensional model of inclinations to drink or not drink. Dashed lines represent factors that promote alcohol avoidance, whereas solid lines represent factors that promote the desire to approach alcohol. This table depicts only the most essential connections with regard to historical factors, expectancies, motivations, and decisions in alcohol use, although other connections may exist.

